# The prevalence of environmentral colonization of Legionella in hospital water systems in Taiwan – a 20 hospital surveillance

**DOI:** 10.1186/1753-6561-5-S6-P240

**Published:** 2011-06-29

**Authors:** YE Lin, YJ Lin, HY Shih, YS Chen

**Affiliations:** 1 National Kaohsiung Normal University, Kaohsiung, Taiwan, China; 2 Kaohsiung Veterans General Hospital, Kaohsiung, Taiwan, China

## Introduction / objectives

Legionnaires’ disease is a major cause of hospital and community acquired pneumonia. Hospital-acquired Legionnaires’ disease is directly linked to the presence of Legionella in hospital drinking water. The objective is to systematically investigate the presence of Legionella and its colonization rate in hospital water systems in Taiwan.

## Methods

Twenty hospitals (Hospitals A to T) throughout Taiwan (8 in northern, 2 in central, 7 in southern, 2 in eastern Taiwan, and one in rural island) were cultured for Legionella. We followed the standardized protocol to perform environmental cultures using (1) water samples; (2) BCYE and DGVP culture media; (3) latex agglutination test (LAX) and direct fluorescent antibody (DFA) technique for *L. pneumophila* speciation and serotyping. We also perform speciation for *L. micdadei* since it is implicated in transplant patients.

## Results

Among 706 water samples collected during 2009 ~ 2011 period, 21% (149/706) were positive for *Legionella*. 65% (13/20) of hospital water systems are positive for Legionella; 2 have >30% site positive, 7 are between 10% ~ 30% site positive, and 4 are <10% site positive. *L. pneumophila* serogroups 1, 3, 5, 6, 7, were isolated from 62% (8/13), 31% (4/13), 8% (1/13), 38% (5/13), and 8% (1/13) of the hospitals, respectively. Five hospitals yielded L. species, but none of them were *L. micdadei*.

## Conclusion

This study allow health official and healthcare professionals for the development of water safety plan to better protect patients and residents of Taiwan in an attempt to prevent Legionnaires’ diseases.

## Disclosure of interest

None declared.

